# Using high-throughput sequencing to investigate the dietary composition of the Korean water deer (*Hydropotes inermis argyropus*): a spatiotemporal comparison

**DOI:** 10.1038/s41598-022-26862-z

**Published:** 2022-12-23

**Authors:** Seung-Kyung Lee, Cheolwoon Woo, Eun Ju Lee, Naomichi Yamamoto

**Affiliations:** 1 School of Biological Sciences, Seoul National University, Seoul, South Korea; 2 Department of Environmental Health Sciences, Graduate School of Public Health, Seoul National University, Seoul, 08826 South Korea; 3 Institute of Health and Environment, Seoul National University, Seoul, South Korea

**Keywords:** Animal behaviour, Conservation biology, Molecular ecology

## Abstract

The Korean water deer (*Hydropotes inermis argyropus*) is considered a vermin in Korea because it damages crops, but also listed as a vulnerable species on the IUCN’s red list. Therefore, it is indispensable to manage them appropriately by understanding the ecology such as food habits. Here, we aimed to apply high-throughput sequencing (HTS), a sensitive and objective method, to investigate the dietary composition of the Korean water deer inhabiting the lowland and forest areas in summer and winter. We targeted the internal transcribed spacer 2 (ITS2) region for plant identification. From a total of 40 fecal samples analyzed, 63 plant genera were identified, with *Morus* being the most abundant, and some of the plant taxa identified by HTS were detected for the first time as the diets of Korean water deer. By type, woody plants (68.6%) were the most predominant, followed by forbs (7.0%) and graminoids (0.7%). We found that the deer in the forest area ate more woody plants (84.6%) than those in the lowland area (52.7%). It was also found that the type of woody plants that the deer ate changed by season. Overall, our results indicate that the Korean water deer is a browser that is seasonally adaptable and feeds on a wide variety of woody plants. We expect that the results and genetics methods reported here, by parallelly investigating their habitat range and reproductive behavior in the future, will help the management and conservation of the Korean water deer, which is in contradictory situations.

## Introduction

The water deer (*Hydropotes inermis*) is a small bodied species in the family Cervidae, and tends to take solitary behaviors^1^. Unlike other Cervidae, the deer lacks antlers and they are characterized by their long canines^2^. The water deer consists of two subspecies inhabiting China (*Hydropotes inermis inermis*) and the Korean peninsula (*Hydropotes inermis argyropus*)^2,3^. Some of the populations were introduced to England and France for hunting game and exhibition purposes^4^ and the arrival of the deer has been reported in Russia recently^5^. The population of the Chinese water deer is declining due to habitat fragmentation and illegal hunting^6^, and the water deer is listed as a vulnerable species on the International Union for Conservation of Nature (IUCN) Red List^6^. However, the Korean water deer inhabits high density from lowlands to forest areas throughout the Korean Peninsula^4^, and the Ministry of Environment of Korea designated it as a vermin because it damages crops^7^. Nonetheless, it is also thought to face serious threats such as habitat changes and roadkills^4^. Therefore, more research (e.g., diet, reproduction, behavior) is needed to take appropriate management and conservation strategies in Korea.

Ruminants are grouped into browsers feeding on plant leaves, grazers feeding on grasses, and the intermediate feeders^8^. The water deer is an intermediate feeder close to a browser^8^. For instance, the Chinese water deer in Zhoushan Archipelago are found to feed on forbs as a main item followed by woods, ferns, and grasses as secondary items^9^. The Korean water deer in Daebu Island are found to mainly feed on forbs or woods^10^. Analysis of the gut contents in the Korean water deer, which died of traffic accidents or natural causes, revealed that forbs were main diets (70%)^11^. Another study conducted in Janghang wetland reported that the Korean water deer fed on forbs (49%), woods (36%), and graminoids (15%)^12^. However, studies conducted in England reported that the captive water deer fed mainly on graminoids and exhibited intermediate characteristics which might be nutritionally suboptimal^13^. Additionally, the deer is more likely to feed on barks or twigs of woody plants in winter season when fresh leaves are not available and increased consumption of woody plants in winter than in summer is observed^9,12^. Thus, habitat vegetation and seasonal differences may have influenced their dietary habits and types^10,12^.

Dietary analysis of wildlife has been traditionally done by examining the feeding traces and/or observing the morphology of gut or fecal contents^14^. However, the problem lies in the need of taxonomic expertise for identifying dietary contents^15^. Recently, high-throughput sequencing (HTS) technologies have been introduced to molecular ecology research^16,17^. These technologies have enabled higher taxonomic resolution^18^ and accurate dietary analysis without the need of special taxonomic expertise^14,19^. Due to its objectivity in species identification and the ability to read a large number of DNA sequences (and therefore high detection sensitivity), the technologies have become widely used in dietary analysis of wildlife, including endangered species^20–24^.

Here, we aimed to apply HTS to investigate the differences in the diet of the Korean water deer by habitats and seasons. Specifically, we collected fecal samples of the water deer in two seasons (summer and winter) in two distinctive habitats (lowland and forest) in Korea, from which DNA was extracted to sequence the internal transcription spacer 2 (ITS2) region of plants. So far, the diet of the water deer has been investigated by feeding sign observation in the field^9,25^, microhistological analysis of the feces^9^, and molecular analyses of the feces, such as polymerase chain reaction-denaturing gradient gel electrophoresis (PCR-DGGE) of the *rbcL* gene^26^ and Sanger sequencing (after cloning) of the *rbcL* gene^10^, ITS2 and *rbcL* gene^12^. In this study, HTS was used to sequence the ITS2 region, which is a primary DNA barcode marker for plants^27,28^. The use of HTS is expected to provide better understandings of feeding habits of the water deer and their seasonal and regional characteristics. We expect that the results of this study will help provide basic information for proper management and conservation strategies for the water deer in Korea.

## Materials and methods

### Study sites and feces sampling

**Figure 1:**
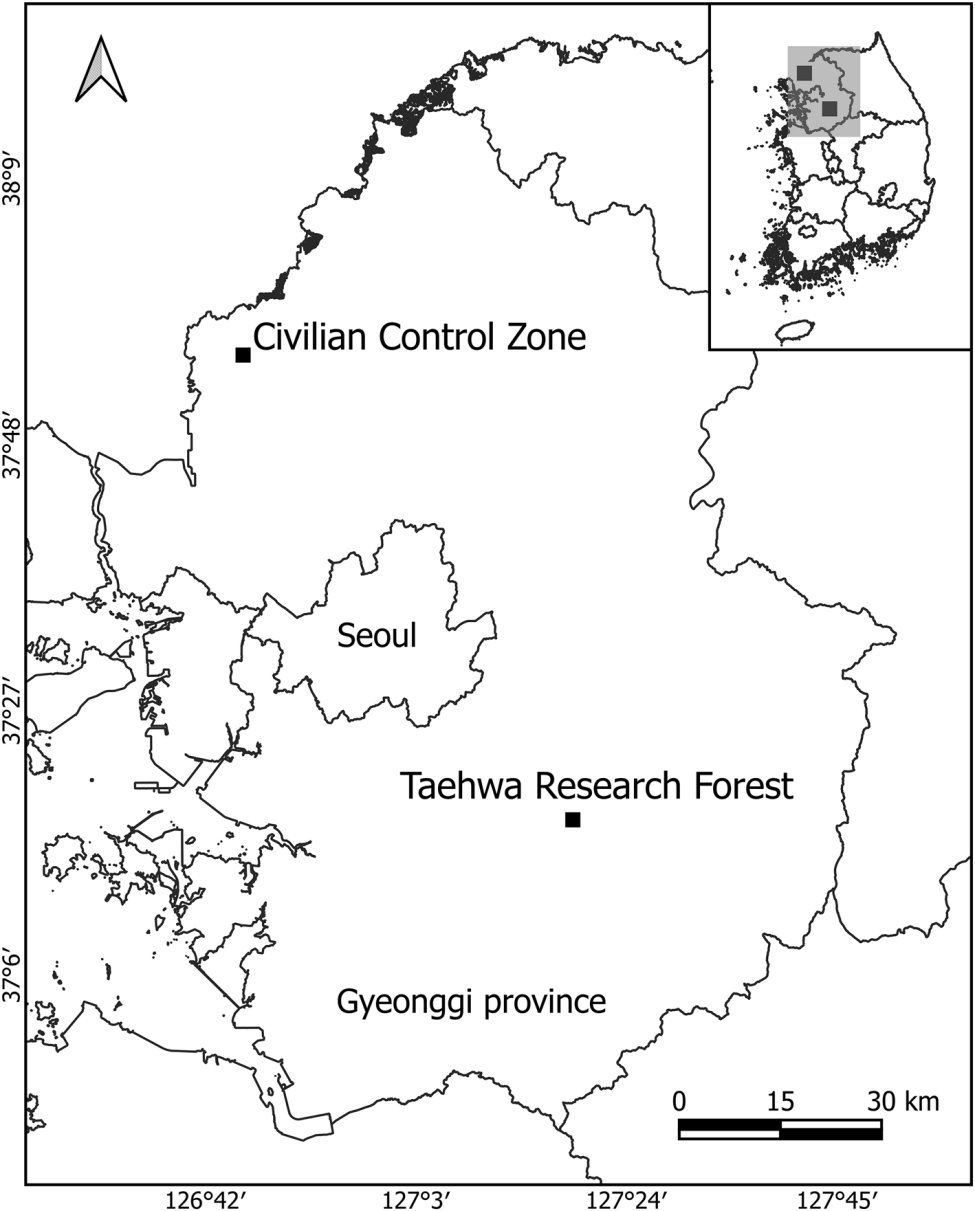
Sampling sites of the Korean water deer feces in Gyeonggi Province, South Korea. The Civilian Control Zone (CCZ) as a representative site of lowland habitats and the Taehwa Research Forest (TRF) as a representative site of forest habitats.

As the Korean water deer inhabit various areas from lowlands to mountainous areas in South Korea^3,10^, we conducted sampling in two different sites to test the diet differences between the habitat types. We selected the Taehwa Research Forest (TRF) as a representative forest for forest habitats and the Civilian Control Zone (CCZ) as a lowland shrub and meadow habitat representative. The elevation of TRF and CCZ is 150–641 m and 0–116 m above sea level respectively. Both sites are in Gyeonggi Province in South Korea (Fig. 1). TRF is an experimental forest administered by Seoul National University, located in Gwangju City, Gyeonggi Province (37°18′34″ N, 127°18′07″ E). TRF consists of both deciduous trees (e.g., *Quercus mongolica* and *Quercus variabilis*) and coniferous trees (e.g., *Pinus koraiensis* and *Larix kaempferi*) artificially planted in the 1970s. The other site CCZ (37°54′56″ N, 126°44′58″E) consists of forest vegetation dominated by *Acer ginnala*, *Alnus japonica*, and *Quercus acutissima*^29^, arable land, and artificial buildings, all of which are typical lowland landscapes of South Korea. As TRF is an experimental forest administered by Seoul National University and the CCZ has restricted access, these areas made for ideal sites for collecting the fecal samples with the fewest amount of human disturbance. The mean annual temperature in 2017 was 10.5 °C for CCZ and 11.7 °C for TRF according to the nearest meteorological observation centers of the Korean Meteorological Administration. Similarly, the total annual precipitation in 2017 was 948 mm in CCZ and 1,020 mm in TRF. The fecal samples were collected in June, July, and August 2017 in the summer, and in December 2017, January and February in 2018 in the winter. In each site in each season, 10 samples were collected, resulting in a total of 40 samples. Each sample consists of several fecal pellets collected from the same pile of fecal pellets. We collected fresh fecal samples that were wet, sticky on the outside. We also avoided sampling after rain and snow. Each of the collected samples was individually placed in a zipper bag, air-dried at room temperature, and stored at − 20 °C^10^.

### DNA extraction

PowerMax Soil DNA extraction kit (Mobio Laboratory, Inc., Carlsbad, CA, USA) was used. From each sample, four fecal pellets were transferred into a conical tube and 4 mL of ultra-pure water was added. Using a sterile spatula, preliminary homogenization was done manually. Then, approximately 0.1 g of each homogenized sample was added to the 2 mL tube of the kit, with the addition of beads (300 mg of 0.1 mm diameter glass beads and 100 mg of 0.5 mm diameter glass beads). The homogenization was performed using a bead beater (BioSpec Products, Inc., Bartlesville, OK, USA) for 3 min at 3,000 g. After that, DNA was extracted as per the manufacturer’s instruction and eluted into 50 µL of TE (10 mM Tris–HCl, 1 mM EDTA, pH = 8.0). The eluted DNA extracts were kept at − 80 °C until the subsequent procedure.

### DNA sequencing

Forty libraries were prepared with plant-specific universal primers of ITS-p3 (YGACTCTCGGCAACGGATA) and ITS-u4 (RGTTTCTTTTCCTCCGCTTA)^30^ to amplify its internal transcribed spacer 2 (ITS2) region. The adapter sequences were attached to universal primers for Illumina MiSeq (Illumina, Inc., San Diego, CA, USA). Each PCR mixture was comprised of a 25 µL of 2 × PCR Solution Premix Taq™ (Takara Bio Inc., Otsu, Shiga, Japan), 1 µL of the DNA extract, and 5 µL of each primer, and 14 µL of PCR-graded water. PCR amplifications were conducted with a T100™ thermal cycler (Bio-Rad Laboratories, Inc., Hercules, CA, USA) and the thermal cycle was as follows: 10 min at 94 °C for initial denaturation, 35 cycles of 30 s at 94 °C, 40 s at 55 °C, 60 s at 72 °C, and 10 min at 72 °C for final extension. The PCR products were purified by AMPure XP beads (Beckman Coulter, Inc., Brea, CA, USA). Then, the purified PCR products were indexed with a Nextera XT Index kit (Illumina) and the thermal cycle was as follows: 3 min at 95 °C for initial denaturation 8 cycles of 30 s at 95 °C, 30 s at 55 °C, 30 s at 72 °C, and 5 min at 72 °C for final extension. The indexed libraries were purified by AMPure XP beads. The indexed and purified libraries were normalized by Quant-iT PicoGreen dsDNA reagent kit (Life Technologies, Carlsbad, CA, USA). The normalized libraries were pooled and loaded to a v3 600 cycle-kit reagent cartridge (Illumina) for 2 × 300 bp paired-end sequencing on an Illumina MiSeq system.

### DNA sequence processing

The raw sequence reads were demultiplexed, and the reads with quality scores of less than 20 were removed by the MiSeq reporter v2.5 (Illumina). The subsequent procedure of sequence assembly, quality check, and assignment of taxonomy was performed using USEARCH v.11.0.667^31^. Reads with more than 1.0 expected errors were removed. The joined reads less than 200 bp were also removed. The default USEARCH settings were used for the identification of unique sequences. Using the UNOISE algorithm, chimeric reads were removed, and the sequence reads were clustered into zero-radius operational taxonomic units (ZOTUs). Each ZOTU was taxonomically assigned against the ITS2 database^32,33^ using the SINTAX algorithm with an 80% confidence level^34^. The ZOTUs with the same taxonomy identification were combined for further taxonomic analysis.

### Data analyses

Prior to the diversity analyses, using the rrarefy function in vegan version 2.5–7^35^, the number of sequence reads in each sample was normalized to the number of sequence reads of the sample from which the lowest number of sequence reads was obtained (i.e., 18,697 reads).

To see whether the Korean water deer has diet selectivity, α diversity analyses were conducted. For α diversity analyses, the rarefaction curves of observed ZOTU richness against the number of sequence reads were created using the iNEXT package version 2.0.20^36^. Alpha diversity indicators such as ZOTU richness, Shannon diversity index, and inverse Simpson index were calculated in vegan. To test for differences in the α diversity indicators between the habitats (forest vs. lowlands) and between the seasons (summer vs. winter), we used a two-way analysis of variance (ANOVA) with habitat and season as fixed factors. Prior to ANOVA, the normality and homoscedasticity of the data were checked and the data were logarithmically transformed if necessary.

To understand the diet composition differences between seasons and sites, we conducted β diversity analyses. For β diversity analyses, the compositional differences between the samples were visualized using nonmetric multidimensional scaling (NMDS) plotting based on the Bray–Curtis dissimilarity using the metaMDS function in vegan. The statistical differences in composition between sites and between seasons were tested with a permutational multivariate analysis of variance (PERMANOVA) with 999 permutations^37^ using the adonis function of vegan. The homogeneity of the compositional dispersion within the same site or within the same season was calculated using the betadisper function of vegan. A variation partitioning analysis was conducted to test the effect size of site and season on variation using the varpart function of vegan. The indicspecies package version 1.7.9^38^ was used to perform the indicator species analysis with 999 permutations to identify plants with different abundances depending on the site or season. To visualize and compare the number of ZOTUs shared between sites and seasons, the Venn diagram illustrating the intersection of the sets of ZOTUs observed at each site and each season was created using the ggvenn package version 0.1.8^39^.

For the diet type analyses, we classified detected plant genera into woody, forb, graminoid, or algae with reference to local flora data (Table S1). Some plants are classified as “not applicable” if the detected genus contains species with different growth forms, or if the taxonomic identification was ambiguous below the 80% confidence level. The local flora data for TRF were adopted from Choi, et al.^40^ and Ko and Shin^41^, Lee, et al.^42^ and those for CCZ were adopted from Kim and Kang^43^ and Gyeonggi Tourism Organization^44^. The differences in the diet type between the sites and between the seasons was statistically tested using the Wilcoxon rank sum test.

All graphs were created using the ggplot2 package version 3.3.5^45^. All analyses were performed on R versions 3.5.3 to 4.0.3^46^. The differences were considered significant if *p* < 0.05.

## Results

### Sequencing results

From a total of 40 libraries, 1,298,966 sequence reads and 1,544 ZOTUs were observed (Table S2). Each library contained 18,697 to 46,475 reads with an average of 32,474 reads. The number of ZOTUs ranged from 55 to 271 per library. The rarefaction curves reached asymptotes for all libraries (Fig. S1), with the sample coverage ranging from 0.9984 to 0.9999 (Table S2). The sampling of sequence reads to the lowest number per library resulted in a total of 747,880 reads and 1544 ZOTUs. After rarefaction, 50 to 264 ZOTUs were identified per library. These ZOTUs were taxonomically classified into 63 genera.

### Taxonomic composition

**Figure 2:**
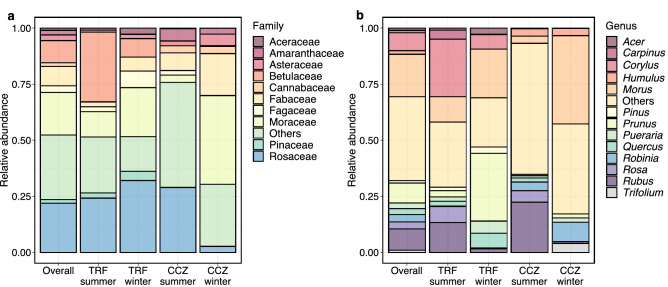
Relative abundance of plants at (**a**) family and (**b**) genus levels. The mean relative abundance of samples in each group is shown. The families and genera with more than more than 1% of relative abundance are shown.

**Figure 3:**
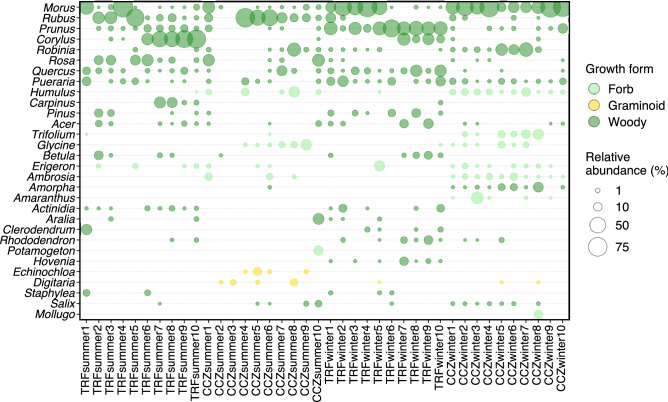
Relative abundance of the 30 most abundant plant genera.

From a total of 1,544 ZOTUs detected, 42 families and 63 genera of plants were identified. The dominant families and their mean relative abundances were Rosaceae (22.0%), Moraceae (19.0%), Betulaceae (10.0%), Fabaceae (8.7%), Fagaceae (2.9%), Asteraceae (2.5%), Amaranthaceae (2.0%), Pinaceae (1.6%), Cannabaceae (1.6%), and Aceraceae (1.0%) (Fig. 2a). At the genus rank, *Morus* (18.9%) was the most dominant, followed by *Rubus* (9.6%), *Prunus* (8.8%), *Corylus* (8.1%), *Robinia* (3.2%), *Rosa* (3.1%), *Quercus* (2.7%), *Pueraria* (2.6%), *Humulus* (1.6%), *Pinus* (1.1%), *Carpinus* (1.0%), *Acer* (1.0%), and *Trifolium* (1.0%) (Figs. 2b and 3). It can be seen that most of the detected genera are present in the local flora (Table S1).

### Indicator species analysis

The indicator species analysis revealed eight indicator genera including *Prunus*, *Quercus*, *Actinidia*, and *Corylus* in TRF, and nine indicator genera including *Humulus*, *Robinia*, *Ambrosia*, and *Glycine* in CCZ (Table 1). All of the eight indicator genera in TRF were woody plants. The indicator genera found in CCZ consisted of forbs, woods, and graminoids. The seasonal analysis revealed two indicator genera of *Rubus* and *Rosa* in summer, and seven indicator genera including *Prunus*, *Amorpha*, *Rhododendron*, and *Hovenia* in winter (Table 2). In general, woody plants were the main indicator genera in both seasons. However, in winter, forbs such as *Trifolium* and *Amaranthus*, as well as graminoids such as *Avena* were also identified as indicator taxa.

**Table 1:** Indicator plant genera identified in each site.

Site	Plant	Association value	*p *value	Frequency		Mean relative abundance (%)		Growth form
				TRF	CCZ	TRF	CCZ	
TRF	*Prunus*	0.942	0.001	95	55	16.51	1.17	Woody
	*Quercus*	0.884	0.002	95	40	4.56	0.99	Woody
	*Actinidia*	0.806	0.001	65	0	1.21	0.00	Woody
	*Corylus*	0.740	0.003	55	15	16.07	0.05	Woody
	*Pinus*	0.703	0.001	50	5	2.04	0.02	Woody
	*Betula*	0.669	0.001	45	5	1.60	0.01	Woody
	*Hovenia*	0.592	0.004	35	0	0.65	0.00	Woody
	*Clerodendrum*	0.500	0.050	25	0	1.18	0.00	Woody
CCZ	*Humulus*	0.866	0.001	10	75	0.00	3.22	Forb
	*Robinia*	0.822	0.008	40	70	0.22	6.25	Woody
	*Ambrosia*	0.806	0.001	0	65	0.00	1.47	Forb
	*Glycine*	0.774	0.001	5	60	0.00	1.86	Forb
	*Salix*	0.730	0.002	10	55	0.02	0.54	Woody
	*Amorpha*	0.707	0.001	0	50	0.00	1.44	Woody
	*Trifolium*	0.592	0.016	5	35	0.00	2.01	Forb
	*Digitaria*	0.547	0.019	5	30	0.00	0.57	Graminoid
	*Acalypha*	0.500	0.048	0	25	0.00	0.46	Forb

**Table 2:** Indicator plant genera identified in each season.

Season	Plant	Association value	*p *value	Frequency		Mean relative abundance (%)		Growth form
				Summer	Winter	Summer	Winter	
Summer	*Rubus*	0.865	0.001	80	30	17.93	1.25	Woody
	*Rosa*	0.740	0.011	55	30	6.12	0.04	Woody
Winter	*Prunus*	0.877	0.005	65	85	1.70	15.98	Woody
	*Amorpha*	0.667	0.005	5	45	0.02	1.42	Woody
	*Rhododendron*	0.647	0.012	10	45	0.08	1.02	Woody
	*Hovenia*	0.592	0.008	0	35	0.00	0.65	Woody
	*Trifolium*	0.548	0.042	10	30	0.00	2.01	Forb
	*Amaranthus*	0.500	0.049	0	25	0.00	1.26	Forb
	*Avena*	0.500	0.036	0	25	0.00	0.05	Graminoid

### Alpha diversity

**Figure 4:**
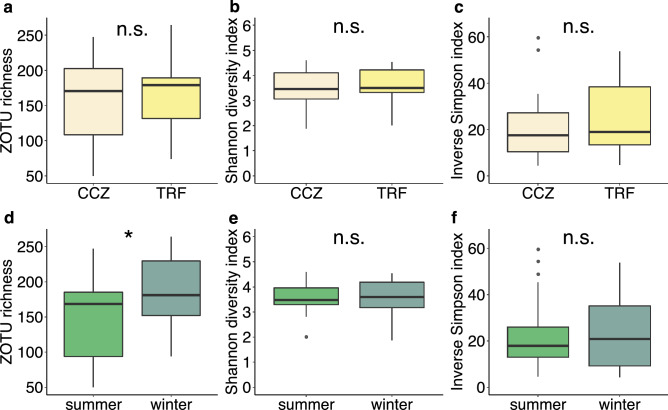
Alpha diversity indices. (**a**) ZOTU richness by site. (**b**) Shannon diversity index by site. (**c**) Inverse Simpson index by site. (**d**) ZOTU richness by season. (**e**) Shannon diversity index by season. (**f**) Inverse Simpson index by season. There was no interaction between sites and seasons (*p* > 0.05; two-way ANOVA). n.s. denotes non-significant and * denotes significant at *p* < 0.05. The rarefied data were used.

ZOTU richness was not significantly different between the sites (*p* > 0.05; two-way ANOVA) (Fig. 4 and Table S3). However, ZOTU richness was significantly higher in winter than in summer with no interaction effect by site (*p* < 0.05; two-way ANOVA). Conversely, the sample-based rarefaction curves demonstrated that ZOTU richness was higher in summer than in winter (Fig. S2). No statistically significant difference in the Shannon diversity index was observed between the sites and between the seasons (*p* > 0.05; two-way ANOVA). Similarly, no statistically significant difference in the inverse Simpson index was observed between the sites and between the seasons (*p* > 0.05; two-way ANOVA).

### Beta diversity

**Figure 5:**
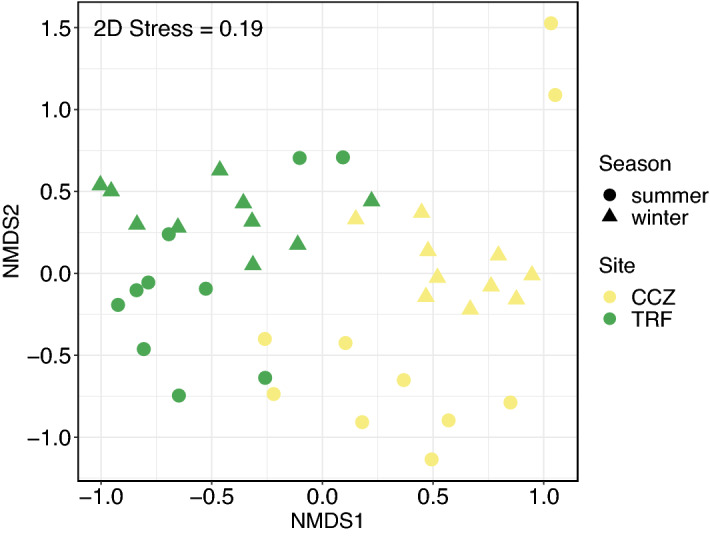
Non-metric multidimensional scaling (NMDS) plot showing the Bray–Curtis dissimilarity based on ZOTU relative abundance.

The distinct differences in plant assemblage structures were observed between the sites and between the seasons (Fig. 5). PERMANOVA test also demonstrated that the seasons explained about 12% of the compositional variance (pseudo-*F* = 5.78; *p* < 0.05, *R*^2^ = 0.120) and that the sites explained about 9% of the compositional variance (pseudo-*F* = 4.23; *p* < 0.05, *R*^2^ = 0.088) (Table S4). The statistically significant interaction between site and season was observed (pseudo-*F* = 2.18; *p* < 0.05, *R*^2^ = 0.045) although it explained only about 5% of the variance.

The variation partitioning analysis shows that the variation by season (*R*^2^_adj_ = 0.13) was larger than the variation by site (*R*^2^_adj_ = 0.03) with no interaction between season and site, suggesting season had greater explanatory power for compositional differences (Fig. S3). Moreover, the beta dispersion analysis shows that the dispersion from the centroid position was significantly higher in summer than in winter (*p* < 0.05; ANOVA), suggesting more diverse diet composition can be seen in summer than in winter (Fig. S4). However, the difference was insignificant between the sites (*p* > 0.05; ANOVA).

The shared ZOTU analysis using the Venn diagram shows that 446 ZOTUs (29%) were shared between the two sites while 507 ZOTUs (33%) and 592 ZOTUs (38%) were found only in the TRF and CCZ samples, respectively (Fig. S5). It also shows that 631 ZOTUs (41%) were shared between the two seasons while 572 ZOTUs (37%) and 342 ZOTUs (22%) were found only in summer and winter, respectively. Seventy-nine ZOTUs (5%) were found in all sites and all seasons. The largest number of site- and season-specific ZOTUs (274 ZOTUs) was observed in CCZ summer, while 177, 135, and 218 ZOTUs were observed uniquely in TRF summer, TRF winter, and CCZ winter, respectively.

### Plant type

**Figure 6:**
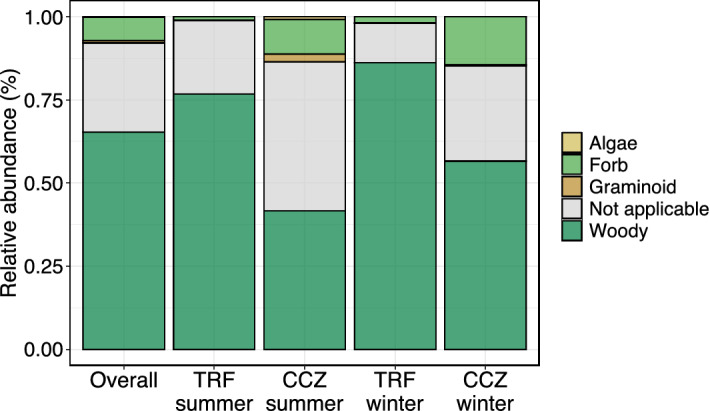
Relative abundance of plants classified by growth morphology. The mean relative abundance of samples in each group is shown.

When plants were classified by growth morphology, the composition was 68.6%, 7.0%, and 0.7% for woods, forbs, and graminoids, respectively (Fig. 6). This trend was consistent across all sites and seasons. However, the relative abundance of each plant type differed between the sites and between the seasons. Specifically, TRF had more woody plants (84.6% of mean relative abundance) than CCZ (52.7%) (*p* < 0.05; Wilcoxon rank-sum test) (Fig. S6). Conversely, CCZ had more forb plants (12.4%) than TRF (1.5%) (*p* < 0.05; Wilcoxon rank-sum test). Similarly, CCZ had more graminoid plants (1.3%) than TRF (0.05%) (*p* < 0.05; Wilcoxon rank sum-test). The comparison between the seasons showed that the only woody plants significantly differed between winter (76.8%) and summer (60.5%) (*p* < 0.05; Wilcoxon rank-sum test).

## Discussion

Understanding the diets of wildlife allows understanding of their ecological role and environmental interactions. In this study, we used HTS to investigate the diet of the Korean water deer, a vulnerable species on the IUCN’s Red List. From a total 40 fecal samples analyzed, we have detected 42 plant families and 63 plant genera. Our HTS-based study detected more plant taxa compared to the results of traditional dietary surveys reported so far for the Korean water deer (Table S5). This is consistent with the results of previous wildlife dietary studies comparing HTS with morphological analysis e.g.,^18,47^. The detected plants included those belonging to the families Rosaceae, Moraceae, Betulaceae, Fabaceae, Fagaceae, Asteraceae, and Amaranthaceae (Fig. 2a). These plants have also been reported as dietary items of the Korean water deer by previous studies using traditional methods^10–12,26,48^. Additionally, we identified, for the first time, plants belonging to the Pinaceae, Araliaceae, Potamogetonaceae, and Apiaceae families in the feces of the Korean water deer.

### The seasonal effects are greater than the habitat effects

Using HTS, this study investigated the seasonal and site differences in the diet composition of the Korean water deer. Overall, we found that the seasons have greater impacts on diet than the sites, in both α (Table S3) and β diversity (Table S4). According to α diversity analysis, the deer seems to have selectivity on favored plant species. The large herbivores generally selectively feed on high-quality forage^49,50^. However, in winter, they consumed more diverse forage than in summer. In winter season, low-quality and availability of forage constrain forage consumption^51^; in response to this, the deer expand their diet range being less selective^52,53^. Meanwhile, as more diverse plant species are available in summer, the sample-based rarefaction curve showed the opposite trend (Fig. S2).

Likewise, the diet composition is mainly affected by season than habitat. The observed tendency appears to be the opposite of that reported in studies investigating other deer species. For example, it has been reported that altitude is the primary factor that affects the dietary differences, and season is secondary for the red deer (*Cervus elaphus*) in a mountainous landscape in New Zealand^54^. Similarly, a review study on the red deer in Europe reports that their diet composition is mainly influenced by habitat rather than season^55^. For the roe deer (*Capreolus capreolus*) in Europe, a review study reports that their diet is more habitat-influenced than seasonal^56^. One explanation for the opposite tendency observed in this study is that the habitat effects relative to the seasonal effects may have been smaller in this study than in other studies. Although we have selected two sampling sites with different landscapes, and the deer have a reported inhabitation range of approximately 2.77 km^2^^57^ and are unlikely to migrate between the two sites, the two sites are relatively close (approximately 83 km in a straight line distance) (Fig. 1). This may have obscured the habitat effects compared to the seasonal effects. Consequently, the differences in the local flora, and thus the feeding habits of the water deer, may have become obscured. Despite the relatively weak habitat effect, it should be noted that this study, as in previous studies, confirmed the distinct difference in the diet composition between the two sites (lowland vs. forest) (Fig. 5 and Table S4).

### The deer in forest areas eat more woody plants than those in lowland areas

The indicator species analysis revealed the plants specifically eaten by the Korean water deer in each site (Table 1). For example, the deer in the forest area (TRF) was found to specifically eat plants belonging to the genera *Prunus*, *Quercus*, *Actinidia*, *Corylus*, and *Pinus*. All of these genera are woody plants and the main constituents in this forest site. Meanwhile, the deer in the lowland area (CCZ) was found to specifically eat plants belonging to the genera *Humulus*, *Robinia*, *Ambrosia*, *Glycine*, *Salix*, *Amorpha*, *Trifolium*, *Digitaria*, and *Acalypha*. Of them, *Humulus*, *Ambrosia*, *Glycine*, *Trifolium*, and *Acalypha* are forbs, and *Digitaria* is a graminoid. These plants are known to be abundant in lowlands and open areas such as the lowland area of CCZ^43^. The analysis of plants by growth morphology also supports the observed tendency. For instance, we found higher proportion of woods in the forest area (TRF) than in the lowland area (CCZ) and higher proportion of forbs and graminoids in the lowland area (CCZ) than in the forest area (TRF) (Figs. 6 and S6). Our observations are consistent with those by Kim et al.^10^ who reported that more forbs (95%) were eaten by the Korean water deer in a lowland area and more woody plants (58%) than forbs (31%) were eaten by those in a mountainous area. These findings make sense because the water deer is known to opportunistically feed on readily available plants^8^.

### The Korean water deer is a seasonally adaptable browser eating a wide variety of woody plants

In addition to the habitat differences, the seasonal differences were also observed. For instance, the proportion of woody plants was higher in winter (76.8%) than in summer (60.5%) in both sampling sites (Figs. 6 and S6). This was also in line with a previous investigation conducted in the Janghang wetland in Korea, by which the increased proportion of woody plants in winter (80%) than in summer (28%) was reported^12^. The increased proportion of woody plants in winter has also been reported in the Chinese water deer^9^. The reported tendency is reasonable since it is well known that the water deer feed on fresh leaves and sprouts in spring and summer, fruits in fall, and twigs, barks and buds of shrubs or trees in winter when fresh leaves are not available^10,12^. It is expected that the consumption of barks and/or twigs in winter greatly contributed to the increase in the proportion of woody plants.

Nevertheless, it was found that woody plants are consumed greatly not only in winter but also in summer (Fig. 6), suggesting that the water deer is a browser by its nature. This is supported by a morphophysiological study, too. For instance, the Chinese water deer introduced to the Whipsnade Zoo Park in England was reported to be predominantly grass-eating due to the lack of woody plants; however, a subsequent investigation suggested that the deer were undernourished due to the unbalanced diets^13^. In addition, although woody plants were predominant in both summer and winter, seasonal differences were observed in the types of woody plants consumed by the deer. For instance, *Rubus* and *Rosa* are found to be preferentially consumed in summer, and *Prunus*, *Amorpha*, *Rhododendron* and *Hovenia* are found to be preferentially consumed in winter (Table 2). From these, the Korean water deer can be regarded as a browser that is seasonally highly adaptable and feeds on a wide variety of woody plants.

### Limitations and future prospects

In this study, we investigated the seasonal and habitat differences of diet composition of the Korean water deer. However, the dietary composition of deer is known to be influenced not only by season and site, but also by other factors, such as gender. For example, female red deer and white-tailed deer (*Odocoileus virginianus*) are known to eat higher quality food than males^58,59^. Conversely, male sika deer (*Cervus nippon*) are known to eat higher-protein diets than females in winter^60^. However, few studies have reported on sex differences in eating habits of the Korean water deer. In future research, in addition to dietary survey, it is desirous to perform sex determination, for example, by analyzing the sex-determining region Y (*SRY*) gene^61^.

Additionally, the gut microbiota of wild animals is related to host species, their diet, and environmental changes such as seasons^62^, and it is also crucial for maintaining health condition of its host^63^. Therefore, analyzing the gut microbiota of wild animals has been proposed as a conservation tool for wildlife, including endangered species^64–66^. In addition to the dietary analysis we have investigated in this study, we propose to analyze the microbiota to contribute to our understanding of the health status of Korean water deer and their protection and management in the future.

In this study, we used DNA barcode sequencing to investigate the dietary composition of the Korean water deer. Though this method is objective and convenient, it also has some drawbacks, as pointed out by previous research^20,67–69^. Most importantly, the parts of plants eaten (e.g., leaves, twigs, bark, and fruits) cannot be differentiated by DNA-based methods. Each part of the plant may have different nutritional values, so an accurate assessment of them is essential to know their ecology. In addition, each part of the plant may have different digestibility^70^. The differences in digestibility cause biases in the proportion of dietary content measured^71–73^. In addition to digestion biases, DNA barcoding can be biased for a variety of reasons, such as variations in the number of cells per plant tissue and the number of copies of the target DNA marker per cell^69^. Recognizing these uncertainties, the results obtained should be used for relative comparisons rather than absolute quantification^19,67^. Depending on the purpose, DNA barcoding may be better to be combined with traditional methods (e.g., morphological observation)^47^.

## Conclusion

In this study, by analyzing fecal samples of the Korean water deer by DNA barcoding, we succeeded in elucidating their dietary tendencies by habitat and season. By using HTS, more plant taxa were detected compared to previous studies, and some taxa were identified for the first time as the diets of Korean water deer. Overall, we found that the Korean water deer is a browser that is seasonally highly adaptable and feeds on a wide variety of woody plants. Diet is associated with animal health, growth, survival, and reproduction^15^. Moreover, understanding their eating habits helps to elucidate their ecological roles, such as their interaction with the environment and their role in the local food web^14,74^, and to formulate conservation strategies such as habitat restoration^19,75^. We expect that our findings and methodologies reported here will be useful for future ecological surveys and for the formulation of their conservation and management strategies. For example, when planning habitat restoration for water deer in the future, it is necessary to restore vegetation with food items that they prefer because restoring vegetation with the target animal’s preferred diet item is suggested^76,77^. Furthermore, for the purpose of reproduction enhancement and population growth, supplementary feeding can be carried out^78^. At the same time, supplementary feeding can be conducted to reduce the damage to arable crops^78,79^. The effectiveness of supplementary feeding seems to relate to the feeding type of the target species^80^, therefore supplying favored forage would be effective. However, supplementary feeding may cause other consequences other than intended management goals^79,81^. Thus, beforehand, it is necessary to assess whether intended consequences can be obtained and consider the comprehensive impacts following supplementary feeding. In addition, in future research, in parallel with the food habits of the wildlife as investigated in this study, it is expected that more accurate conservation and management strategies will be established by investigating the ecology of the target wildlife, such as their habitat range and reproductive behavior.

## Supplementary Information

Supplementary Information.

## Data Availability

Raw sequence data are available at NCBI under the accession number PRJNA791828.
