# Supplementary material for: Using high-throughput sequencing to investigate the dietary composition of the Korean water deer (Hydropotes inermis argyropus): a spatiotemporal comparison
Source: Sci Rep. 2022 Dec 23;12:22271. doi: 10.1038/s41598-022-26862-z (PMC9789119; doi:10.1038/s41598-022-26862-z)
Supplement: Supplementary file 1 — Supplementary Information. [file 41598_2022_26862_MOESM1_ESM.pdf]

Supporting information of:

Using high-throughput sequencing to investigate the dietary composition of the Korean water deer (*Hydropotes inermis argyropus*): A spatiotemporal comparison

Seung-Kyung Lee[1], Cheolwoon Woo[2], Eun Ju Lee[1], Naomichi Yamamoto[2,3]*

[1]School of Biological Sciences, Seoul National University, Seoul, South Korea

[2]Department of Environmental Health Sciences, Graduate School of Public Health, Seoul National University, Seoul, South Korea

[3]Institute of Health and Environment, Seoul National University, Seoul, South Korea

* Corresponding author

Department of Environmental Health Sciences, Graduate School of Public Health, Seoul National University, Seoul 08826, South Korea

Phone: +82-2-880-2837

E-mail: nyamamoto@snu.ac.kr

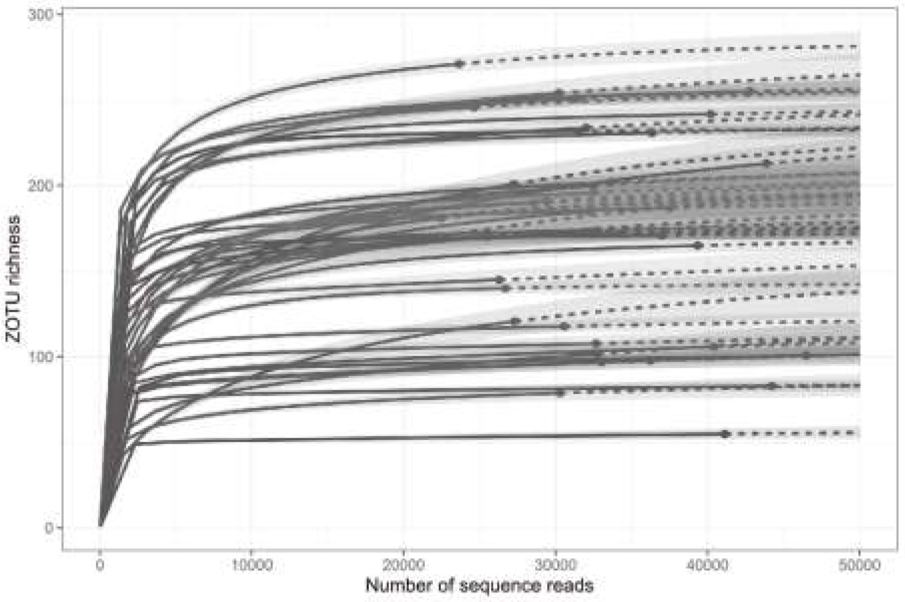

Fig. S1. Rarefaction curves of observed ZOTU richness against the number of sequence reads.

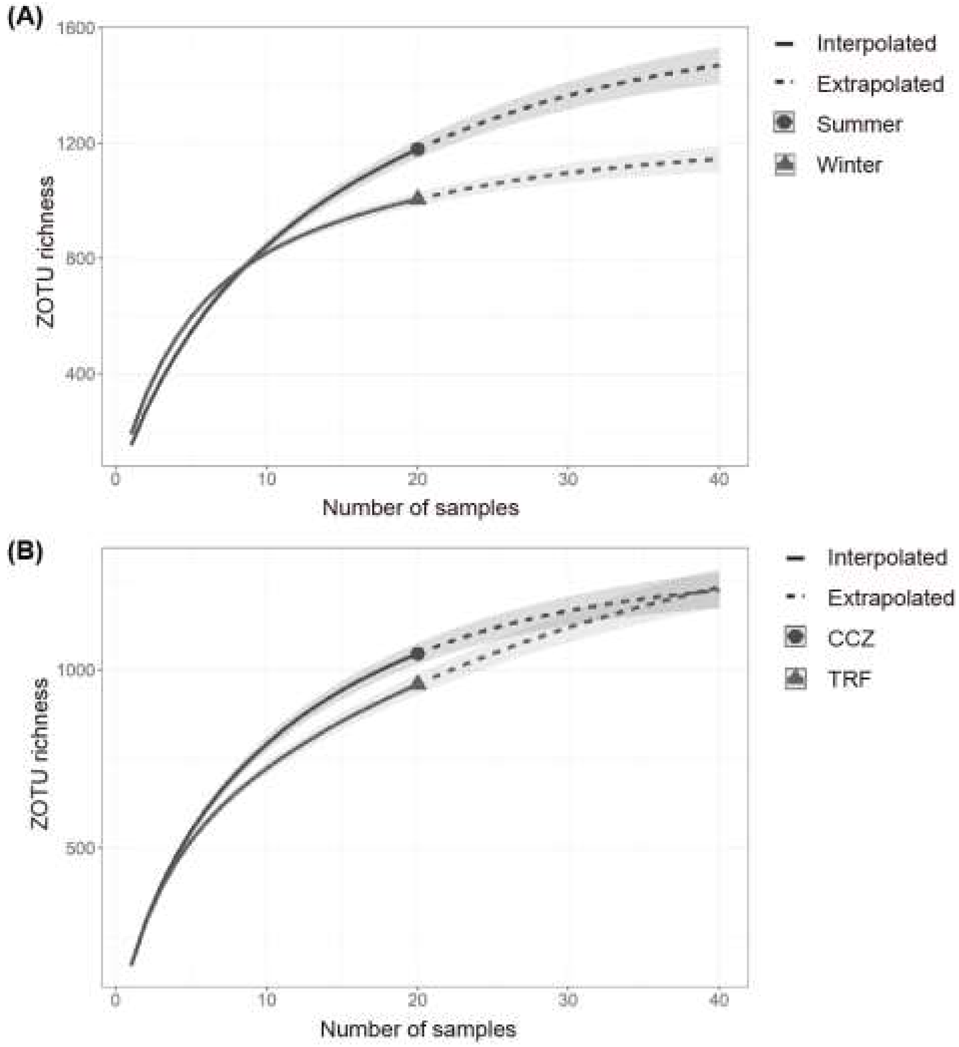

Fig. S2. Sample-based rarefaction curve for season (A) and site (B).

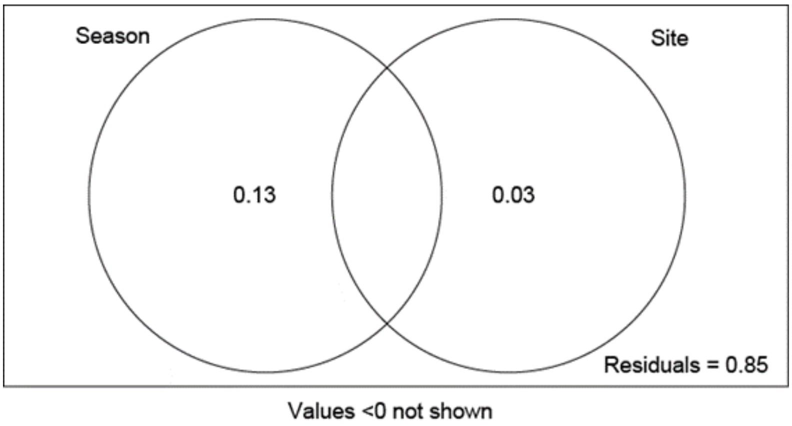

Fig. S3. Variation partitioning analysis for seasons and sites.

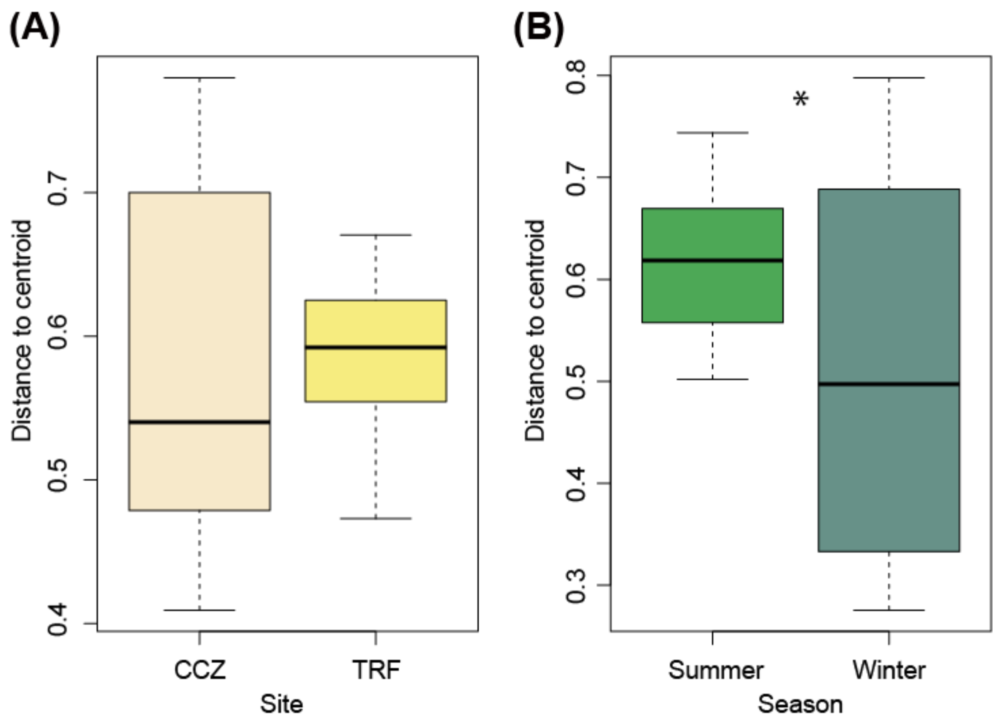

Fig. S4. The result of betadisper analysis, homogeneity of the dispersion between sites and between seasons. (*, $p < 0.05$).

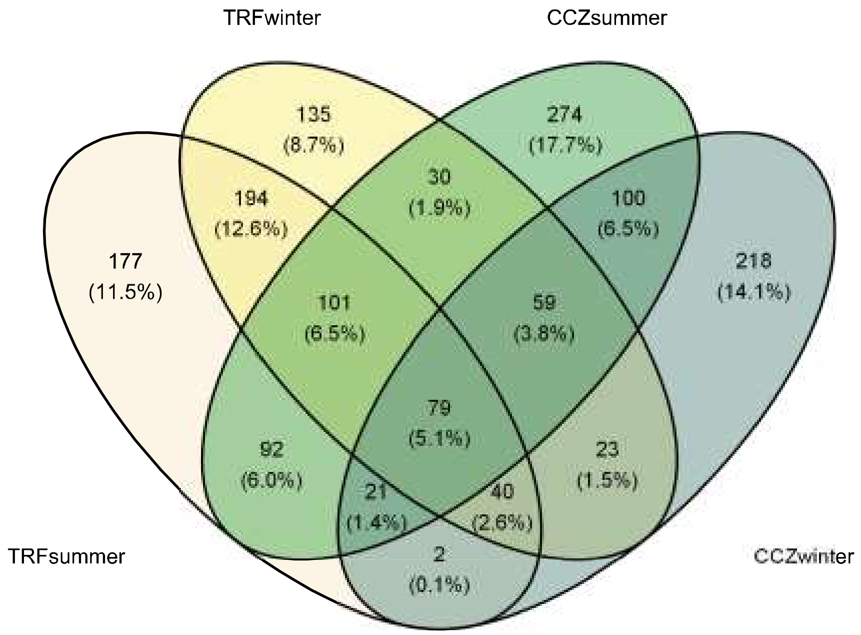

Fig. S5. Venn diagram showing the number of shared ZOTUs.

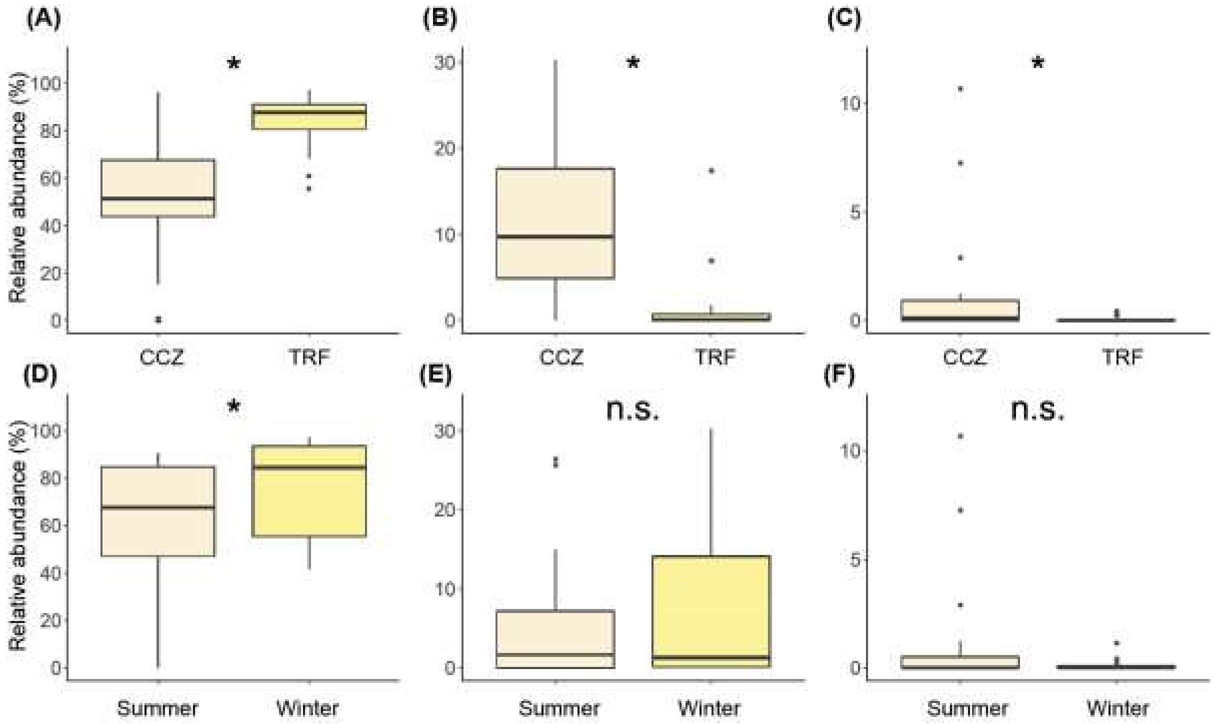

Fig. S6. Relative abundance of growth form compared between site and season. (A) woody species (B) forb species (C) graminoid species compared between site (CCZ and TRF). (D) woody species (E) forb species (F) graminoid species compared between season (summer and winter). (*, $p < 0.05$; Wilcoxon rank-sum test).

Table S1. List of plant species that inhabit each site. Of a total 451 genera (all genera found from CCZ and TRF), 189 genera (41.9%) are shared in both CCZ and TRF, 237 genera (52.5%) are unique in CCZ, and 25 genera (5.5%) are unique in TRF.

Genus	Type	TRF [a]	CCZ [b]
Acalypha	Forb	*Acalypha australis*	*Acalypha australis*
Acer	Woody	*Acer palmatum*; *Acer pictum* var. *mono*; *Acer pseudosieboldianum*; *Acer tataricum* subsp. *ginnala*	*Acer buergerianum*; *Acer palmatum*; *Acer pictum* var. *mono*; *Acer pseudosieboldianum*; *Acer tataricum* subsp. *ginnala*; *Acer palmatum* var. *dissectum*
Actinidia	Woody	*Actinidia arguta*; *Actinidia kolomikta*; *Actinidia polygama*	
Ainsliaea	Forb	*Ainsliaea acerifolia*	
Alnus	Woody		*Alnus incana* subsp. *hirsuta*; *Alnus japonica*
Amaranthus	Forb	*Amaranthus blitum* subsp. *oleraceus*; *Amaranthus tricolor*	*Amaranthus blitum* subsp. *oleraceus*; *Amaranthus tricolor*
Ambrosia	Forb		*Ambrosia artemisiifolia*; *Ambrosia trifida*
Amorpha	Woody		*Amorpha fruticosa*
Ampelopsis	Woody	*Ampelopsis heterophylla*	*Ampelopsis heterophylla*
Angelica	Forb	*Angelica anomala*; *Angelica dahurica*; *Angelica decursiva*	*Aneilema keisak*; *Angelica cartilaginomarginata*; *Angelica cartilaginomarginata* var. *distans*; *Angelica dahurica*; *Angelica decursiva*; *Angelica gigas*; *Angelica polymorpha*
Aralia	Woody	*Aralia elata*	*Aralia cordata* var. *continentalis*; *Aralia elata*
Arenaria	Forb		*Arenaria serpyllifolia*
Avena	Graminoid		*Avena fatua*; *Avena sativa*
Betula	Woody	*Betula davurica*; *Betula pendula*; *Betula schmidtii*	*Betula davurica*; *Betula pendula*
Bracteacoccus	Algae		
Brassica	Forb		*Brassica juncea*
Carpinus	Woody	*Carpinus cordata*; *Carpinus laxiflora*	*Carpinus laxiflora*
Cerastium	Forb		*Cerastium glomeratum*; *Cerastium holosteoides* var. *hallaisanense*
Chamaecrista	Forb	*Chamaecrista nomame*	*Chamaecrista nomame*
Chlorosarcinopsis	Algae		
Clerodendrum	Woody	*Clerodendrum trichotomum*	*Clerodendrum trichotomum*
Cocculus	Woody	*Cocculus orbiculatus*	*Cocculus orbiculatus*
Conyza	Forb		*Conyza canadensis*
Corylus	Woody	*Corylus heterophylla*	*Corylus heterophylla*
Dactylis	Graminoid		*Dactylis glomerata*
Desmodesmus	Algae		
Digitaria	Graminoid	*Digitaria ciliaris*	*Digitaria ciliaris*

Echinochloa	Graminoid		*Echinochloa crus-galli*; *Echinochloa crus-galli* var. *echinatum*
Erigeron	Forb		*Erigeron annuus*; *Erigeron philadelphicus*
Forsythia	Woody		*Forsythia koreana*
Fraxinus	Woody	*Fraxinus rhynchophylla*; *Fraxinus sieboldiana*	*Fraxinus mandshurica*; *Fraxinus rhynchophylla*; *Fraxinus sieboldiana*
Glycine	Forb		*Glycine max* subsp. *soja*
Hovenia	Woody		*Hovenia dulcis*
Humulus	Forb	*Humulus scandens*	*Humulus scandens*
Ipomoea	Forb		*Ipomoea lacunosa*; *Ipomoea nil*; *Ipomoea purpurea*; *Ipomoea triloba*
Isodon	Forb	*Isodon inflexus*	*Isodon excisus*; *Isodon inflexus*; *Isodon japonicus*
Klebsormidium	Algae		
Lespedeza	NA	*Lespedeza bicolor*; *Lespedeza cyrtobotrya*; *Lespedeza virgata*	*Lespedeza bicolor*; *Lespedeza cuneate*; *Lespedeza cyrtobotrya*; *Lespedeza davurica*; *Lespedeza juncea*; *Lespedeza maximowiczii*; *Lespedeza pilosa*; *Lespedeza thunbergii* subsp. *formosa*; *Lespedeza tomentosa*; *Lespedeza virgata*
Ligustrum	Woody	*Ligustrum obtusifolium*	*Ligustrum obtusifolium*
Lysimachia	Forb	*Lysimachia clethroides*; *Lysimachia barystachys*	*Lysimachia barystachys*; *Lysimachia clethroides*; *Lysimachia coreana*; *Lysimachia vulgaris* var. *davurica*
Medicago	Forb		*Medicago sativa*
Mollugo	Forb		*Mollugo pentaphylla*; *Mollugo verticillata*
Morus	Woody	*Morus australis*	*Morus alba*; *Morus australis*; *Mosla dianthera*
Neillia	Woody		*Neillia uekii*
Oplismenus	Graminoid	*Oplismenus undulatifolius*	*Oplismenus undulatifolius*
Phryma	Forb	*Phryma leptostachya* var. *oblongifolia*	*Phryma leptostachya* var. *oblongifolia*
Phyllanthus	Forb		*Phyllanthus ussuriensis*
Pinus	Woody	*Pinus densiflora*; *Pinus koraiensis*; *Pinus rigida*	*Pinus densiflora*; *Pinus koraiensis*; *Pinus rigida*; *Pinus strobus*
Potamogeton	Forb		*Potamogeton distinctus*
Potentilla	Forb	*Potentilla freyniana*; *Potentilla fragarioides*	*Potentilla discolor*; *Potentilla fragarioides*; *Potentilla freyniana*; *Potentilla kleiniana*; *Potentilla supine*; *Potentilla supina* var. *ternata*
Prunus	Woody	*Prunus japonica* var. *nakaii*; *Prunus serrulata* f. *spontanea*; *Prunus serrulata* var. *pubescens*; *Prunus padus*; *Prunus persica*; *Prunus sargentii*; *Prunus armeniaca*; *Prunus glandulosa*	*Prunus* × *yedoensis*; *Prunus armeniaca*; *Prunus davidiana*; *Prunus japonica* var. *nakaii*; *Prunus mume*; *Prunus padus*; *Prunus persica*; *Prunus sargentii*; *Prunus serrulata* f.

			spontanea; *Prunus serrulata* var. *pubescens*; *Prunus spachiana* f. *ascendens*; *Prunus tomentosa*; *Prunus subhirtella*
Pueraria	Woody	*Pueraria lobata*	*Pueraria lobata*
Quercus	Woody	*Quercus acutissima*; *Quercus aliena*; *Quercus dentate*; *Quercus mongolica*; *Quercus serrata*; *Quercus variabilis*	*Quercus acutissima*; *Quercus aliena*; *Quercus dentata*; *Quercus mongolica*; *Quercus serrata*; *Quercus variabilis*; *Quercus palustris*
Rhododendron	Woody	*Rhododendron mucronulatum*; *Rhododendron schlippenbachii*; *Rhododendron mucronulatum* var. *ciliatum*	*Rhododendron indicum*; *Rhododendron mucronulatum*; *Rhododendron schlippenbachii*; *Rhododendron yedoense* f. *poukhanense*
Robinia	Woody	*Robinia pseudoacacia*	*Robinia pseudoacacia*
Rosa	Woody	*Rosa multiflora*	*Rosa multiflora*
Rubus	Woody	*Rubus crataegifolius*; *Rubus parvifolius*	*Rubus corchorifolius*; *Rubus crataegifolius*; *Rubus hirsutus*; *Rubus ikenoensis*; *Rubus parvifolius*; *Rubus pungens*
Salix	Woody	*Salix caprea*; *Salix gracilistyla*; *Salix pierotii*	*Salix caprea*; *Salix gracilistyla*; *Salix koriyanagi*; *Salix pierotii*; *Salix pseudolasiogyne*; *Salix triandra* subsp. *nipponica*; *Salix xerophila*
Staphylea	Woody	*Staphylea bumalda*	*Staphylea bumalda*
Symplocos	Woody	*Symplocos sawafutagi*	*Symplocos sawafutagi*
Tilia	Woody		
Trifolium	Forb	*Trifolium repens*	*Trifolium pratense*; *Trifolium repens*
Wisteria	Woody		*Wisteria floribunda*

[a] Choi, et al. [1], Ko and Shin [2] and Lee, et al. [3].
[b] Kim and Kang [4] and Gyeonggi Tourism Organization [5].

Table S2. Sampling site and season for each sample, their number of sequence reads, ZOTU richness, ZOTU richness after the rarefaction to minimum number of sequence reads i.e., 18697 reads per sample.

No.	Sample ID	Site	Season	Sequence Reads	ZOTU richness	Sample Coverage Estimate	ZOTU richness after rarefaction
1	TRFsummer1	Taehwa Research Forest	Summer	40383	106	0.9997	99
2	TRFsummer2	Taehwa Research Forest	Summer	32472	196	0.9996	189
3	TRFsummer3	Taehwa Research Forest	Summer	37464	187	0.9998	180
4	TRFsummer4	Taehwa Research Forest	Summer	30248	79	0.9997	74
5	TRFsummer5	Taehwa Research Forest	Summer	32984	97	0.9997	93
6	TRFsummer6	Taehwa Research Forest	Summer	32623	108	0.9998	104
7	TRFsummer7	Taehwa Research Forest	Summer	22421	181	0.999	178
8	TRFsummer8	Taehwa Research Forest	Summer	29375	191	0.9994	185
9	TRFsummer9	Taehwa Research Forest	Summer	30175	254	0.9993	247
10	TRFsummer10	Taehwa Research Forest	Summer	28512	190	0.9996	187
11	CCZsummer1	Civilian Control Zone	Summer	36922	171	0.9999	170
12	CCZsummer2	Civilian Control Zone	Summer	31468	232	0.9999	228
13	CCZsummer3	Civilian Control Zone	Summer	34280	172	0.9998	165
14	CCZsummer4	Civilian Control Zone	Summer	41087	55	0.9999	50
15	CCZsummer5	Civilian Control Zone	Summer	46475	101	0.9998	94
16	CCZsummer6	Civilian Control Zone	Summer	27185	201	0.9985	186
17	CCZsummer7	Civilian Control Zone	Summer	32325	173	0.9996	167
18	CCZsummer8	Civilian Control Zone	Summer	36179	98	0.9997	92
19	CCZsummer9	Civilian Control Zone	Summer	44180	83	0.9999	79
20	CCZsummer10	Civilian Control Zone	Summer	32660	174	0.9998	171
21	TRFwinter1	Taehwa Research Forest	Winter	31974	234	0.9995	227
22	TRFwinter2	Taehwa Research Forest	Winter	26640	140	0.9998	137
23	TRFwinter3	Taehwa Research Forest	Winter	25432	170	0.9992	167
24	TRFwinter4	Taehwa Research Forest	Winter	32428	200	0.9994	191
25	TRFwinter5	Taehwa Research Forest	Winter	19978	167	0.9984	165
26	TRFwinter6	Taehwa Research Forest	Winter	30512	118	0.9998	115
27	TRFwinter7	Taehwa Research Forest	Winter	18697	180	0.9992	180
28	TRFwinter8	Taehwa Research Forest	Winter	24600	246	0.9994	241
29	TRFwinter9	Taehwa Research Forest	Winter	26237	145	0.9996	143
30	TRFwinter10	Taehwa Research Forest	Winter	23586	271	0.9992	264
31	CCZwinter1	Civilian Control Zone	Winter	36282	231	0.9998	228
32	CCZwinter2	Civilian Control Zone	Winter	33622	202	0.9996	194
33	CCZwinter3	Civilian Control Zone	Winter	43840	213	0.9992	182
34	CCZwinter4	Civilian Control Zone	Winter	32159	185	0.9993	171
35	CCZwinter5	Civilian Control Zone	Winter	40151	242	0.9998	234
36	CCZwinter6	Civilian Control Zone	Winter	42768	255	0.9998	247
37	CCZwinter7	Civilian Control Zone	Winter	39287	165	0.9998	155
38	CCZwinter8	Civilian Control Zone	Winter	31364	251	0.9996	240
39	CCZwinter9	Civilian Control Zone	Winter	27280	121	0.9989	113
40	CCZwinter10	Civilian Control Zone	Winter	32711	102	0.9994	94
Total				1,298,966	1544		1544

Table S3. Two-way analysis of variance table for site (TRF and CCZ) and season (summer and winter) on alpha diversity indices (ZOTU richness, Shannon diversity, Inverse Simpson index). Statistically significant effects are highlighted in bold. Inverse Simpson index data were log-transformed data to meet the assumption.

	d.f.	ZOTU richness			Shannon diversity index			Inverse Simpson index		
		Mean Square	F-value	Significance	Mean Square	F-value	Significance	Mean Square	F-value	Significance
Site	1	27.225	0.009	0.924	0.215	0.39	0.536	96.853	0.395	0.533
Season	1	15563.025	5.226	0.028	0.00009386	0	0.99	0.721	0.003	0.957
Site x Season	1	1265.625	0.425	0.519	0.817	1.483	0.231	449.466	1.835	0.184
Error	36	2978.108			0.551			244.912		

Table S4. PERMANOVA table for the comparison between site and season.

	df	F.Model	R2	Pr(>F)	
Site	1	4.234	0.08785	0.002	**
Season	1	5.7752	0.11983	0.001	***
Site x Season	1	2.1843	0.04532	0.026	*
Residuals	36	0.31747	0.74699		
Total	39		1		

Table S5. Previous and current studies on diet content of the Korean water deer.

Method		Sample type/method	Primer for DNA analysis	Sampling information		Number of sample/duration of survey	Identified taxa			Reference
				Number of sites	Season		Family	Genus	Species	
DNA analysis	PCR-DGGE	Rumen content	*rbcL*	≥2 [a]	Sp, Su, F, W	29(17) (successfully PCR)	6			Park, et al. [6]
	Sanger sequencing	Feces	202 bp *rbcL*	2	Su	20(15) (successfully PCR)	24			Kim, et al. [7]
	Sanger sequencing	Feces	ITS2 + *rbcL*	1	Sp, Su, F, W	77	20	35		Kim, et al. [8]
	High-throughput sequencing	Feces	ITS2	2	Su, W	40	42	63		**This study**
Direct or field sign observation	Rumen content observation	Rumen content		≥2 [a]	Sp, Su, F, W	44	18	11	12	Park, et al. [9]
	Field sign methods	Feeding sign		1	Sp, Su, F, W	22 times of day survey	21	37	41	Lee [10]
	Feeding sign survey/hearing investigation	Feeding sign/by asking farmer (in case of agricultural plant)		7	From April to October	From April 2007 to October 2010	27	49	62	Kim [11]

[a] Sampling site information is not available for some samples.

Supplementary References

1 Choi, D.-Y. *et al.* Flora of province Gyonggi-do. *Bull. Seoul Nat'l Univ. Arbor.* **21**, 25–76 (2001).

2 Ko, S. & Shin, Y. Flora of middle part in Gyeonggi Province. *Korean J. Plant Res.* **22**, 49–70 (2009).

3 Lee, S.-K., Ryu, Y. & Lee, E. J. Endozoochorous seed dispersal by Korean water deer (*Hydropotes inermis argyropus*) in Taehwa Research Forest, South Korea. *Glob. Ecol. Conserv.* **40**, e02325 (2022).

4 Kim, K.-H. & Kang, S.-H. Flora of western civilian control zone (CCZ) in Korea. *Korean J. Plant Res.* **32**, 565–588 (2019).

5 Gyeonggi Tourism Organization. Pyeonghwa-Nuri Trail ecological resource survey. (Paju City, Gyeonggi Province, Korea, 2018).

6 Park, J.-E., Kim, B.-J. & Lee, S.-D. A study of potential of diet analysis in the Korean water deer (*Hydropotes inermis argyropus*) using polymerase chain reaction-denaturing gradient gel electrophoresis (PCR-DGGE). *Korean J. Environ. Ecol.* **24**, 318–324 (2010).

7 Kim, B. J., Lee, N. S. & Lee, S. D. Feeding diets of the Korean water deer (*Hydropotes inermis argyropus*) based on a 202 bp *rbcL* sequence analysis. *Conserv. Genet.* **12**, 851–856 (2011).

8 Kim, J., Joo, S. & Park, S. Diet composition of Korean water deer (*Hydropotes inermis argyropus*) from the Han River Estuary Wetland in Korea using fecal DNA. *Mammalia* **85**, 487–493 (2021).

9 Park, J.-E., Kim, B.-J., Oh, D.-H., Lee, H. & Lee, S.-D. Feeding habit analysis of the Korean water deer. *Korean J. Environ. Ecol.* **25**, 836–845 (2011).

10 Lee, B. *Morphological, ecological and DNA taxonomic characteristics of Chinese water deer (Hydropotes inermis Swinhoe).* Ph.D. thesis. Chungbuk National University (2003).

11 Kim, E.-K. *Behavioral ecology, habitat evaluation and genetic characteristics of water deer (Hydropotes inermis) in Korea.* Ph.D. thesis. Kangwon National University (2011).
